# Mimicking blood and lymphatic vasculatures using microfluidic systems

**DOI:** 10.1063/5.0175154

**Published:** 2024-05-06

**Authors:** Eva Hall, Kailee Mendiola, N. Keilany Lightsey, Donny Hanjaya-Putra

**Affiliations:** 1 Aerospace and Mechanical Engineering, Bioengineering Graduate Program, University of Notre Dame, Notre Dame, Indiana 46556, USA; 2 Department of Chemical and Biomedical Engineering, The University of Texas at San Antonio, Texas 78249, USA; 3 Chemical and Biomolecular Engineering, University of Notre Dame, Notre Dame, Indiana 46556, USA

## Abstract

The role of the circulatory system, containing the blood and lymphatic vasculatures, within the body, has become increasingly focused on by researchers as dysfunction of either of the systems has been linked to serious complications and disease. Currently, *in vivo* models are unable to provide the sufficient monitoring and level of manipulation needed to characterize the fluidic dynamics of the microcirculation in blood and lymphatic vessels; thus *in vitro* models have been pursued as an alternative model. Microfluidic devices have the required properties to provide a physiologically relevant circulatory system model for research as well as the experimental tools to conduct more advanced research analyses of microcirculation flow. In this review paper, the physiological behavior of fluid flow and electrical communication within the endothelial cells of the systems are detailed and discussed to highlight their complexities. Cell co-culturing methods and other relevant organ-on-a-chip devices will be evaluated to demonstrate the feasibility and relevance of the *in vitro* microfluidic model. Microfluidic systems will be determined as a noteworthy model that can display physiologically relevant flow of the cardiovascular and lymphatic systems, which will enable researchers to investigate the systems' prevalence in diseases and identify potential therapeutics.

## INTRODUCTION

The circulatory system, comprised of the blood and lymphatic vascular systems, in the human body is essential for the maintenance of cell homeostasis, which includes cell growth/development, absorption of nutrients within cells, and removal of waste from cells.^1^ The blood vascular system moves oxygenated blood through the arteries from the heart, exchanges nutrients and solutes at the capillaries, and returns blood to the heart through the veins.^1,2^ The lymphatic vascular system transports lymph (i.e., fluid leaked from blood capillaries that contain cells, proteins, and macromolecules, which are found in the interstitial space) through lymphatic capillaries to collecting vessels and the lymph nodes (LNs) and then back to the blood vascular system.^2,3^ Although the functionalities of each system are distinct, dysfunction of either can lead to serious complications and disease.^4^

The prominent role of the circulatory system to its surrounding tissues and the whole body has created an increased desire for researchers to investigate its impact on many diseases. More specifically, researchers have been trying to develop *in vitro* models that can replicate the sensitive blood and lymph movement in the circulatory system.^5^ As *in vivo* circulatory models are unable to provide high resolution imaging observations of the blood and lymphatic micro-vessels, *in vitro* models can provide an easier platform to not only monitor the circulatory environment but also develop and manipulate the system biomimetically.^5^ Furthermore, *in vivo* circulatory models are limited in assessing accurate physical quantities and determining accurate flow properties as demonstrated and discussed in Koutsiaris *et al.* when they identified the high variability of their findings and previous work.^6^ With respect to practical feasibility, the use of large-scale animal models that could replicate the circulatory system in humans is costly and requires more care.^7^ Although small animal models are cost-effective and low maintenance, replication of the human blood and lymphatic systems is limited by their differing sized and physiology, which makes disease modeling difficult to mimic.^7^

A promising *in vitro* model that can replicate the desired physiological conditions of the cardiovascular and lymphatic systems is a microfluidic device.^8^ These devices can demonstrate the normal behavior of fluids within micro-vessels as well as the impact of various mechanical, chemical, biological, and electrical factors.^9^ As a growing model in circulatory research, there are a variety of microfluidic fabrication methods available to develop a more advanced device including both blood and lymphatic vessels.^9,10^ One recent fabrication method includes the patterning of porous polyethylene terephthalate (PET) membranes on poly (dimethylsiloxane) (PDMS) substrates to create channels for endothelial cells (ECs) to investigate permeability in microcirculation.^5^ Another recent fabrication method involved the use of bioprinting to form the required vessel-like tubing of the blood and lymphatic systems within a multi-layered PDMS/poly(methyl methacrylate) (PMMA) microfluidic substrate, which contained a hydrogel chamber for tumor replication to investigate these circulatory systems in response to tumor cells.^11^ Current methods of vascular cell sourcing (stem-cell derived, endothelial cells, etc.) and biomaterial approaches (natural, synthetic, etc.) can also be combined and further explored to stimulate fluid flow in microfluidic devices.^12^

**FIG. 1.**
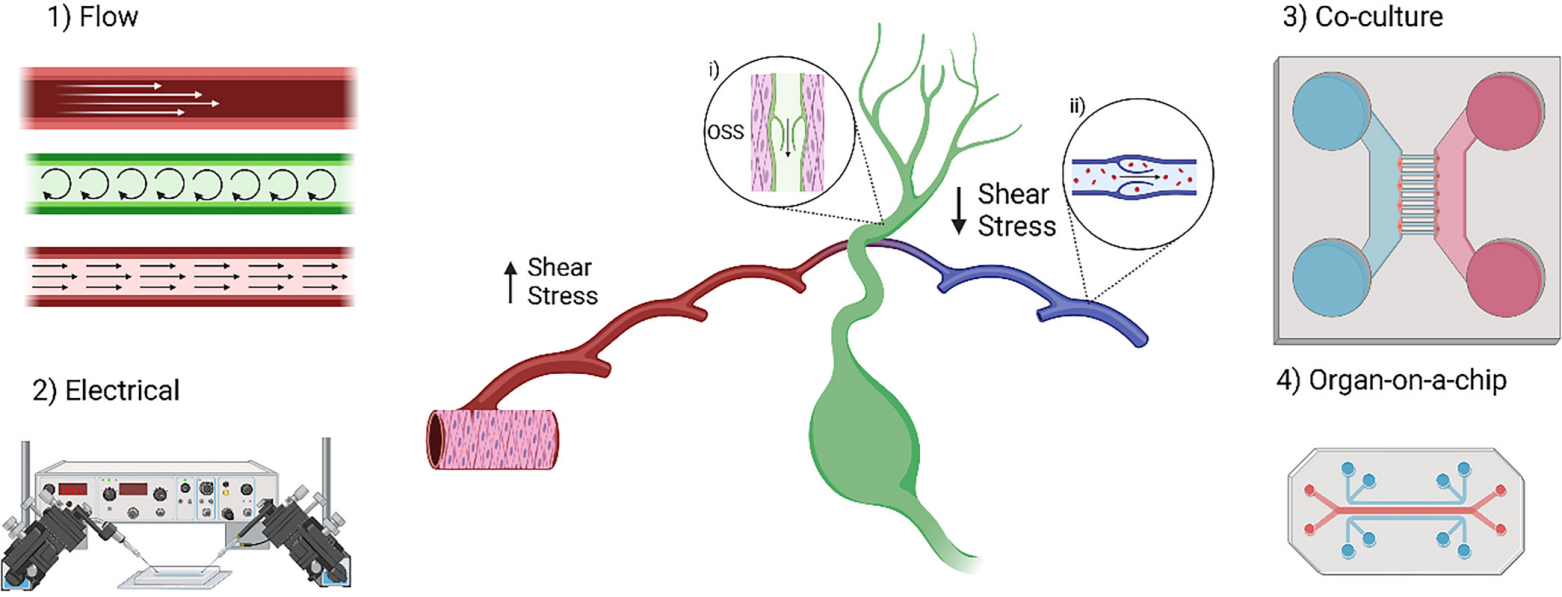
Microfluidics can be used in many types of systems with different types of vasculatures. Devices have been used to create various physiologically relevant models that integrate flow and electrical communication. In addition, co-culture and organ-on-a-chip systems have been used to explore intercellular communication and develop more complicated models. The lymphatic vessels are made of lymphatic endothelial cells (LECs). The capillaries have discontinuous button junctions making them able to take up cells, lymph, and solutes. The pre-collecting and collecting vessels have continuous zipper junctions and are surrounded by lymphatic muscle cells (inset i). These muscles contract to drive lymph through the valves, causing oscillatory flow and applying oscillatory shear stress (OSS) to the surrounding endothelial cells. The blood vasculature has smooth muscle running circumferentially around vessels to move blood. The blood vessels experience high shear stress, while the veins, venules, and lymphatic vessels experience low shear stress. The veins also have valves to prevent backflow of de-oxygenated blood (inset ii). Created with BioRender.com.

The evaluation and manipulation of fluid flow in a model is desired for the investigation of the circulatory system because the forces from the flow of blood and lymph within their respective systems affect cell/vessel behavior and function.^13,14^ One of the main forces of interest is shear stress, which is defined as a frictional force parallel to the cells lined within the blood and lymphatic vessels.^14^ Shear stress is critical for determining normal physiological behavior in vessels, which highlights the importance of recognizing its characteristics (Fig. 1).^15,16^ Another factor to consider is flow type, either laminar or non-laminar, as shear stress is directly proportional to flow which means changes in flow can impact how the cells within the vessel behave.^14–19^ While both are directly impacted by fluid flow, it is important to note that each system contains different cells (i.e., blood ECs and lymphatic endothelial cells, LECs) and is its own entity. Thus, each system experiences different flow types and shear stresses, which will invoke differing responses between the systems.^20^ When developing a microfluidic device for the circulatory system or determining the impact of the circulatory system on disease, it is important to consider these environmental and behavioral differences and how they impact tissue/system development. Hence, the design criteria for these microfluidic devices have been heavily influenced by the study of vascular biology and its implementation in a variety of engineering disciplines.^21^

Table I summarizes the important mechanical behaviors and corresponding cellular responses to the flow types and shear stress features associated with normal physiological blood and lymphatic systems that have been observed over a range of varying studies. These characteristics should be considered when designing or investigating the circulatory system. A key difference that is noted between the two systems is the principal flow type experienced by each—the vascular system mostly experiences pulsatile flow while the lymphatic system is primarily oscillatory. Though both systems will generally experience both pulsatile and oscillatory flow, each system will mostly demonstrate its principal flow type. Another important consideration is that the blood system generally experiences a higher range of shear stress rates while the lymphatic system generally experiences a lower magnitude and range of shear stress rates. With respect to mechanotransduction signaling, both systems prefer anti-inflammatory environments (i.e., no inflammatory cytokines, etc.) and further facilitate an anti-inflammatory EC response. However, in response to flow type, the blood ECs respond to pulsatile flow by elongating and orienting toward the flow while the LECs undergo morphological changes for valve development.

**TABLE I.** Comparison of the mechanical and cell behavioral characteristics found in the two circulatory systems.

Characteristics	Blood vascular system^a^	Lymphatic vascular system^b^	References^c^
Flow type	Pulsatile (laminar)	Oscillatory (non-laminar)	14,17,18
Shear stress rates^d^	High (1−80 dyne/cm^2^)	Low (0.1−12 dyne/cm^2^)	15–17,20,22
Inflammatory response	Anti-inflammation	Anti-inflammation	15,18
Endothelial cell behavior	Elongated, oriented toward flow	Morphological changes for valve development	14,16,18,19

^a^ System includes arteries, arterioles, veins, venules, and capillaries at all sizes (i.e., macro- and micro-molecular).

^b^ System includes initial lymphatic vessels, collecting lymphatic vessels, and capillaries.

^c^ References are previous studies in which the flow/cell behavior of these systems were characterized in some type of manner and/or briefly touched upon during experimentation. The methodologies and major findings within these studies are outside of the scope of this review.

^d^ General range of values from the various components of the systems.

In this review, the increased complexity of the circulatory system within the body will be discussed to highlight the need for an approachable model to investigate relevant processes. More specifically, the fluid flow present in the blood and lymphatic vessels will be described in detail with reference to relevant physiological functions within the systems to explain specific force mechanisms. Additionally, the use of calcium cations as intracellular communicators in both the endothelial and muscle cells within the blood and lymphatic systems will be discussed to highlight how flow impacts electrical behavior. Then, an evaluation of the use of co-culturing cells for the physiological replication of the blood and lymphatic systems in different applications will be presented to demonstrate the feasibility and relevance of the *in vitro* microfluidic model. Organ-on-a-chip models that display fluid flow in relevant blood and lymphatic vessel cell types will also be referenced to moreover prove its expanding interest in medicine. The study of the circulatory system and its role in pathologies is expected to expand in research as the identification of the relationship between blood, lymph, and other fluids in the body becomes more apparent. Microfluidics displaying the physiologically relevant flow of the cardiovascular and lymphatic vasculatures will enable researchers to investigate its prevalence in diseases and identify potential therapeutics.

## FLOW

Flow within the lymphatic and vascular vessels is crucial to understanding physiological conditions including atherosclerosis, lymphedema, and cancer. For blood vasculature, flow is stimulated by the contraction of the heart, while the lymphatic system relies on the anatomy of the vessels for the propulsion of fluid flow. By understanding the structures within the vasculature, the conditions that need to be replicated for experimentation become comprehensible.

**FIG. 2.**
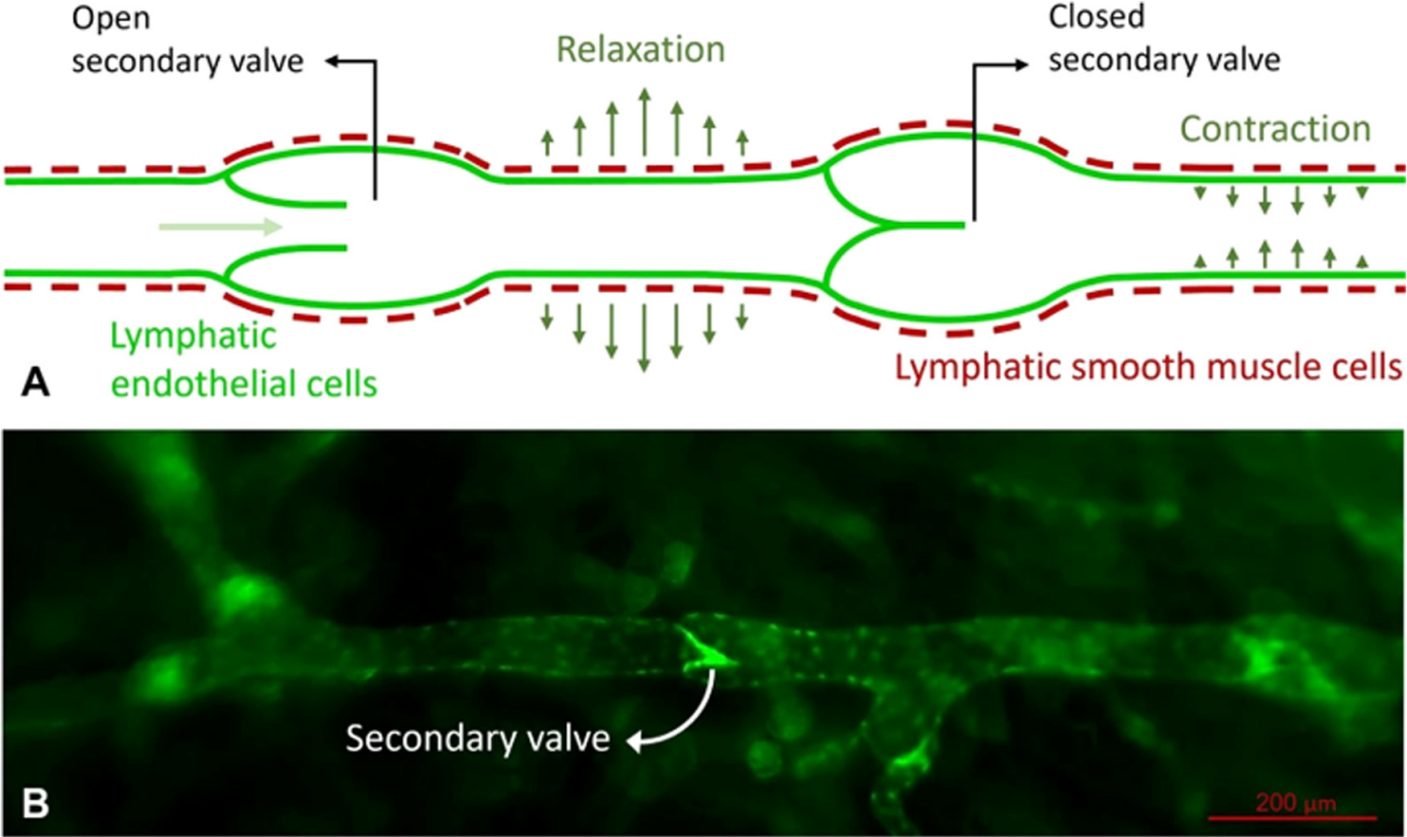
Lymphatic vasculature consists of multiple singular functional units called lymphangions. Lymphangions comprise a valve and channel that allow the unit to act as both pump and conduit for fluid flow. (a) Diagram of the lymphangion while undergoing both functions and (b) the fluorescent image of lymphatic vessels using Prox^1^-GFP mouse both communicate the anatomy and physiology of the vessel. Both images are adapted from Koudehi *et al*.^23^

Lymphatic vessels are comprised of numerous functional singular units, termed lymphangions. A lymphangion consists of a one-way valve, lymphatic muscle cells (LMCs), and a layer of endothelial cells lining the inside of the lumen (Fig. 2).^18,19,23^ The way these units operate is by a spontaneous depolarization of the pacemaker cells belonging to the lymphatic muscle cells generating an action potential, the action potential causes the lymphangion to contract increasing the intravascular pressure and causing the closure of the input valve and opening of output valve causing the lymph to flow through.^24^ The lymphangions propel the fluid to flow in two main convulsive modes: passive, tonic contractions, and short phasic tensing.^22,25^ The units alternate between these two modes individually based on mechanical stimuli, which demonstrates that flow properties can differ in the same system depending on the region.^25^

Unlike the lymphatic system, blood circulation is not comprised of a singular functional unit as it can consist of arteries, arterioles, capillaries, and other structures that belong to the venous sections.^26^ As the system is not broken down into units, while studying fluid flow in these structures the scope being looked at varies depending on the experiment. Initiation of circulation instigates EC growth and is influenced by the level of oxygen as well. To study this in-depth, there have been microfluidic devices designed to look at the influence of flow and oxygen on endothelial colony-forming cells (ECFCs) and endothelial cells derived from human induced pluripotent stem cells.^27,28^

**FIG. 3.**
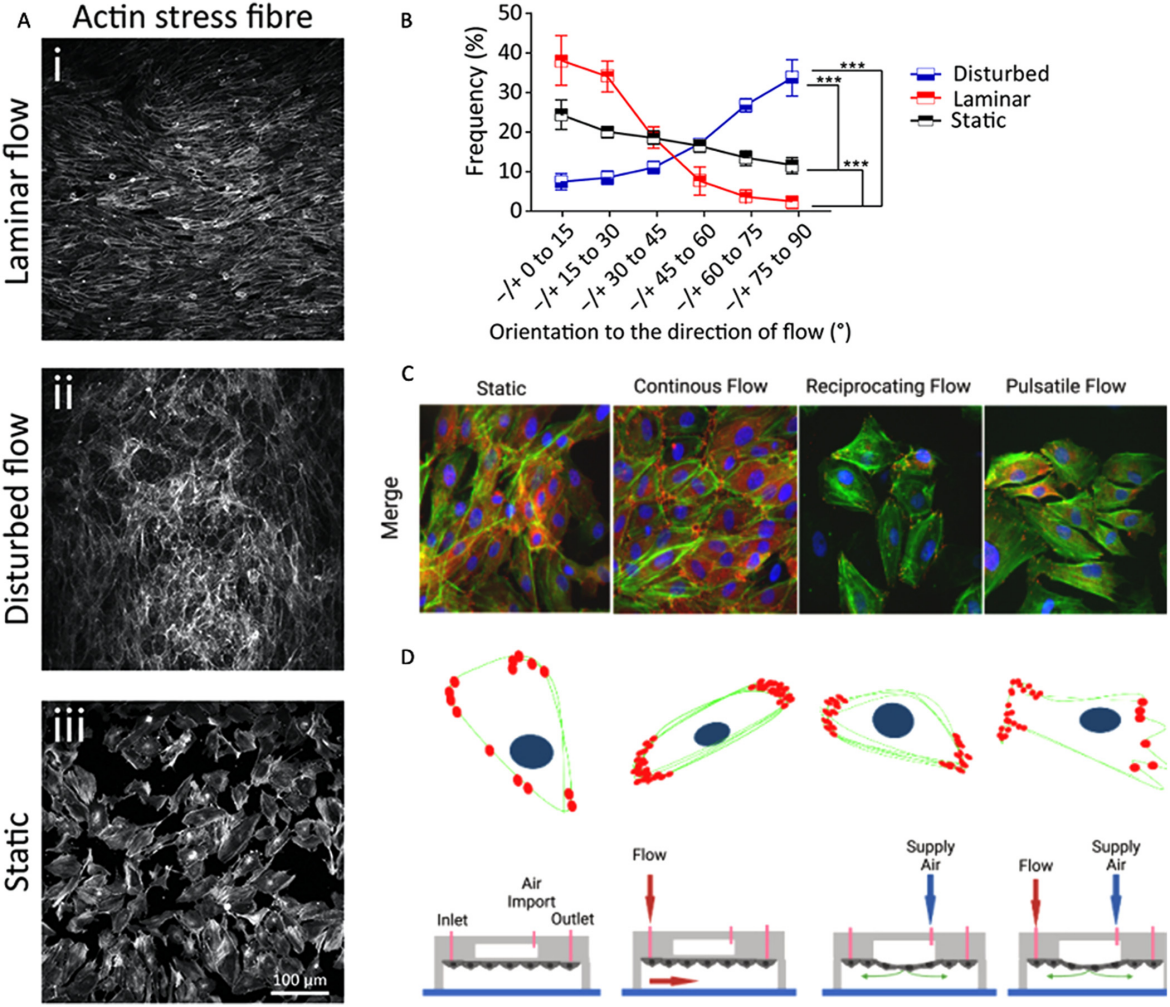
Microfluidic devices allow the introduction of flow while having control overflow characteristics. This figure indicates how these devices can be used to investigate the effects of different flow characteristics on endothelial cell morphology, orientation, and physiology. (a) and (b) Endothelial cell (HAECs) orientation comparison after introduction of different flow patterns via microfluidic device designed by Tovar-Lopez *et al.*^29^ (c) and (d) Superimposed fluorescent images of Bovine Aortic Endothelial Cells (BAECs) looking at the nucleus, F-actin, and paxillin by Chu *et al.* presents how the cell physiology differs under different flow fields.^30^

There are three main forces that both types of vessels encounter *in vivo* that factor into fluid flow: circumferential (hoop), axial, and shear stress [Fig. 3(a)]. The mechanical stressors that the vessels sustain can affect the outcomes of fluid transport by different mechanosensory mechanisms [Fig. 3(b)]. Hoop stress is the result of transmural pressure radiating outward in all directions of the vessel causing stress in the circumferential direction. When transmural pressure is increased, the level of fluid transport increases by upregulating C–C motif chemokine ligand 21 (CCL21) and intracellular adhesion molecule 1 (ICAM-1) and the downregulation of platelet endothelial cell adhesion molecule-1 (PECAM-1) and vascular endothelial cadherin (VE-Cadherin).^31^ Shear stress is a direct result of the fluid flowing along the direction of the vessel wall and is one of the main physical mechanisms coordinating the pumping action between lymphangia as a response to the mechanical microenvironment. The imposed shear stress stimulates the mechanism known as shear-dependent inhibition. For this mechanism, the lymphangion transitions between the pump and conduit for fluid flow as the shear stress within the vessel is inversely related to contraction.^25^

Flow not only affects the entire lymphangion or lumen but also the ECs lining the vessels. These cells have mechanosensitive factors that also play a role in fluid flow for both vessel types. Transmural flow increases fluid transport while also supporting vascular endothelial migration and encourages vascular sprouting during angiogenesis. A study found sprouts emerged in regions with nominal shear stress in vessels with reduced pressures.^32^ Shear stress within the units stimulates mechanosensors within the cells that activate the enzyme endothelial nitric oxide synthase (eNOS). This enzyme in turn releases nitric oxide, which is a known vasodilator by decreasing the chance for spontaneous depolarization needed for the generation of action potentials to induce contraction.^5,33,34^

To replicate flow for these two systems *in vitro*, researchers have developed microfluidic pump systems to facilitate flow affecting a variety of parameters such as flow profiles or flow type. Dixon *et al.* utilized a microfluidic pump system consisting of a vessel chamber from Living Systems Instrumentation and a perfusion set that allows for control of both the circumferential stress and shear stress independently.^25,35^ The system is set up with two syringes that operate independently by a solenoid valve switching the inlet and outlet pumps that as one reaches empty the valve automatically switches to the other. The flow remains unidirectional and pressure from the flow is recorded at two points, the inlet and outlet, which uses the information gathered for a feedback loop to keep the pressure gradient constant.^35^

Developing an environment that allows for the control of flow properties can enable the comparison of blood and lymphatic EC behavior. Alternative methods to manipulate flow characteristics are modifications to the microfluidic channels within the chips. Chen *et al.* found that combining a Y-shaped device that has a rectangular-shaped mixing channel alongside a microfluidic channel with varying cross sections allows observations of two different variables that affect the cell, flow, and ATP calcium signaling, both together and separately [Fig. 3(d)].^36^ Another study manipulated characteristics of the microfluidic channels to observe the effects of disturbed flow on ECs, human aortic endothelial cells (HAECs), by patterning the channel.^29^

## ELECTRICAL

Microfluidic devices can be used to observe intercellular communication. Detection of intercellular communication is conducted through observation of the electrical charges through internal concentrations of ions. Recently, calcium has become an ion of interest due to its role in indicating the EC function. When the cells transmit information between each other, their internal concentration of calcium fluctuates. Noren *et al.* found that there are three patterns of internal calcium fluctuations that indicate different cellular characteristics: null response, repeated spikes, and low persistence.^37^ When the calcium concentration undergoes no changes, the pattern is determined to be a null response and is not designated into a cell phenotype unlike the repeated spikes or the low persistence.

The repeated spiking pattern is when the internal calcium concentration increases and decreases dramatically multiple times within a specific timeframe. The repeated spiking pattern is associated with proliferative qualities as the cells that experienced a higher level of activated Nuclear Factor of Active T-cells 2 (NFAT2). One study found that adjusting the amount of shear stress on LECs can make the internal cell calcium concentration pulse, similar to what was seen of proliferative cells in Noren *et al.*, only that the frequency of pulses was sensitive to the magnitude of the shear stress.^38^ Understanding the role of calcium and proliferation has led to advances in the modeling of calcium interactions in relation to endothelial cells within microfluidic devices. One study was able to predict calcium responses to the surrounding microenvironment using mathematical models, another experiment was able to generate a 3D model of endothelial cells within a microfluidic device looking at the release of calcium and its effects on the endothelial cells.^39,40^

The dynamics of how these cells communicate is important to understand as the cells may use cations differently. One study found that ECs transmit calcium signals in a direction determined by the areas of cell polarization. After creating a microfluidic device with a micropatterned surface, they influenced the shape and direction of the ECs [Fig. 3(c)].^41^ For LECs, the calcium dynamic mechanisms are optimized for depolarization while vascular ECs are enhanced for hyperpolarization, which has opposite effects on the vessels' contraction, dilation, and constriction, respectively.^42^ To affect the vessels' contraction, the system uses calcium signaling to communicate information from EC to EC as well as EC to muscle cells as the vascular walls of both the vascular and lymphatic systems comprise both types of cells. To understand the relation between singular cell lines and co-cultures of the cells during calcium signaling, many studies have microfluidic systems in conjunction with organ-on-chip technologies.^43^

To study the communication methods between the endothelial cells, cell pairing aids in optimization for observing cell–cell interactions. There are various cell-pairing methods using microfluidic devices such as hydrodynamic methods, microwells, electricity-assisted methods, etc.^43,44^ Yin *et al.* fabricated a dielectrophoretic-microfluidic device for both stimulation and observing interactions between cells during cell pairing.^45^ Another experiment used dielectrophoretic cell pairing methods for observing intercellular communications is advantageous compared to other methods as it maintains cell integrity and can be more meticulous in its organization in microfluidic devices.^46,47^

## CO-CULTURE

Many systems have been developed with biomaterials, such as collagen,^48^ hyaluronic acid,^49,50^ and fibrin,^51,52^ to encourage the growth of 3D vessels. However, they are missing the cell interactions that co-cultures provide, some of which are outlined in Table II. With the addition of microfluidics to co-culture, a more physiologically relevant model may be achieved. For example, Bachmann *et al.* utilized adipose derived stem cells and human umbilical vein endothelial cells (HUVECs) in a hydrogel to look at the effect of growth factor concentration on vessel formation and maturation by utilizing microfluidics to create a spatiotemporal gradient.^53^ A circular chamber was attached to the side of the channel where media were flowed through, providing indirect interstitial flow. By changing the diameter of the chamber, the researchers found a diffusion distance limitation for the growth factors. To compare the indirect to direct flow, the media flowed through the center of the hydrogel. Indirect flow of growth factors increased sprouting, while direct perfusion caused more cell alignment, but less sprouting.

**TABLE II.** Co-culture and organ-on-a-chip models.

System	Endothelial cells	Additional cells	Flow	Application	References
Co-culture	HUVECs	Adipose derived stem cells	Yes	Effect of growth factor concentration gradients and flow on vessels.	53
	Human aortic vein ECs	Human aortic smooth muscle cells	Yes	Effect of VSMCs on Aortic ECs.	54
	Human dermal microvascular ECs	Mammary adenocarcinoma cells, human glioblastoma cell line, and smooth muscle precursor cells	Yes	Effect of cancer cells and VSMCs on EC migration.	55
	Human retinal microvascular ECs	Pericytes	Yes	Effect of flow on pericyte and EC co-cultures.	56
	HUVECs	Endometrial stroma	Yes	Modelling endometrial tissues	57
	Brain ECs	Glial cells	Yes	Neuroactive drug testing on BBB vessels.	58
	iPS derived ECs and HUVECs	Embryonic Stem Cell derived Motor Neurons	Yes	Calcium signaling in an EC–neuron spheroid model.	59
	LECs	Fibroblasts	Yes	Effect of fibroblast paracrine factors on lymphatic vessel formation.	60
	LECs	PBMCs	Yes	PBMC–LEC interactions with applied interstitial flow	61
	Dermal microvascular ECs and lymphatic microvascular ECs	N/A	Yes	LEC–BEC microvasculature model	5
	Immortalized human microvascular ECs	Human breast carcinoma cell line	Yes	Shear stress effects on EC-Tumor model	62
	Human cerebral microvascular ECs	Adenocarcinoma, squamous cell carcinoma, and human brain astrocyte	Yes	Brain Tumor microenvironment model	63
	LECs	Healthy and tumor derived fibroblasts	No	Effect of tumor derived fibroblasts on lymphatic vasculature	64
	LECs, iPS derived ECs, and HUVECs	Healthy and breast cancer associated fibroblasts	No	Effect of breast cancer associated fibroblasts on vasculature	65
	HUVECs and LECs	Breast cancer cells	Yes	Effect of Breast cancer on lymphatic and blood vasculature	66
	Human brain microvascular ECs	Pericytes and astrocytes	Yes	Blood brain barrier model	67
Organ-on-a-chip	Human cerebral microvascular ECs	Neural progenitor cells	No	Alzheimer's brain model	68
	HUVECs	Fibroblasts and retinal pigmented epithelial cells	No	Outer blood retinal barrier model	69
	HUVECs	Fibroblasts and retinal pigmented epithelial cells	Yes	Outer blood retinal barrier model	70
	Human capillary microvascular ECs	Intestinal epithelial cell and colonoid-derived epithelial cells	Yes	Human gut model	71
	LECs	Lymphatic muscle cells	Yes	Lymphangion Model	72
	LECs	N/A	Yes	Effects of VEGF-A, VEGF-C, and flow on lymphatic vessels	73
	LECs	N/A	Yes	Effects of acute and chronic inflammation on lymphatic vessel permeability and drainage.	74
	LECs	Blood ECs	Yes	Effects of ROCK inhibition on LECs and blood ECs	75
	HUVECs	Mouse pancreatic cancer cells	No	Adenocarcinoma remodeling endothelium	76
	Immortalized HUVECs	Fibroblasts, lung cancer cells, ovarian cancer cells, kidney cancer cells, and CAR-T cells	Yes	Perfusable vascularized tumor spheroids	77
	Immortalized LECs	Mouse colon cancer cells	No	Effect of colon cancer on lymphatic sprouting	78
	LECs	Breast cancer cells	Yes	Effect of breast cancer/CCL21 on lymphatic sprouting	79
	HUVECs and LECs	Breast cancer cells	Yes	LEC–BEC–tumor interface	11
	Dermal microvascular ECs and LECs	Melanoma cells and fibroblasts	No	LEC–BEC–melanoma interface	80

Since arteries are surrounded by vascular smooth muscle cells (VSMCs), many researchers have investigated EC–VSMC interactions in co-culture systems. Engeland *et al.* created a microfluidic chip with two parallel channels separated by a PDMS membrane allowing for contact between ECs and VSMCs.^54^ ECs were exposed to flow to mimic hydrodynamics seen in the vessel wall while cyclic strain like what is seen during pulsatile blood flow was provided by two vacuum pumps. The cells grown in the chip were able to be maintained for 4 days, expressed the expected markers and morphology, and exhibited a slight increase in Notch signaling. Other groups have looked at the EC–VSMC interactions through paracrine signaling. Chung *et al.* created a microfluidic system with three parallel channels with scaffolding between them to allow for EC migration.^55^ ECs were seeded into the central channel with control media on one side and various other cell types including VSMCs and cancer cells on the other. This system allowed the researchers to observe migration through 3D matrices and to determine how the factors generated by the other cell lines influence migration. Some of the cancer lines increased EC attraction and sprouting, while the VSMCs decreased EC migration. Overall, these studies allow us to gain more insight into EC–VSMCs interactions and develop platforms that provide hydrodynamics conditions, which can be used for more angiogenesis studies in the future.

Another cell type that interacts with ECs *in vivo* is pericytes, a cell type that encompasses the outside of the vessel.^81^ Rogers *et al.* integrated ECs and pericytes in a high throughput microfluidic system called PREDICT96.^56^ This system contained a microporous membrane allowing for EC–pericyte interactions while providing cell type specific readouts. The device was able to keep the cells viable for up to 14 days, worked with permeability and barrier disruption assays, and allowed the cells to be imaged. Luminex and PCR were also able to be done on both the EC and pericyte channels. After shear stress was applied to the EC channel, an increase in pro-inflammatory cytokine IL-6 was seen. This platform may be helpful for future studies with its high throughput, multilayered design, and integration of flow.

**FIG. 4.**
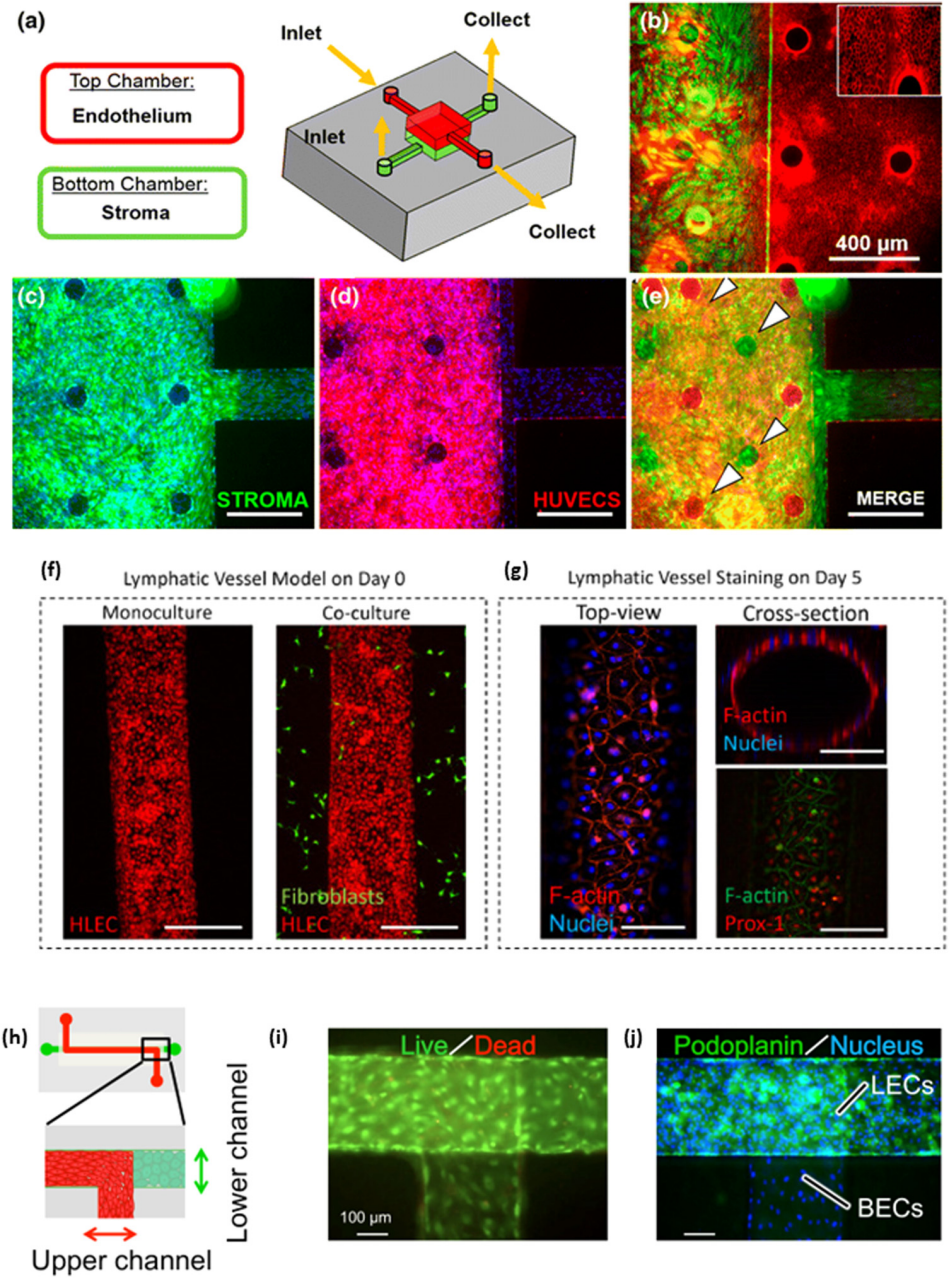
Co-culture systems for blood and lymphatic endothelial cells. (a) A model for a co-culture system with human umbilical vein cells (HUVECs) and stromal cells. A membrane between the two allows for the study of paracrine signaling without cell–cell interactions between the HUVECs and the stroma. (b) Characterization of morphology of stromal cells and HUVECs. Stromal cells were stained with vimentin (green) in the lower chamber. HUVECs were stained with CD31 (red). The inset is ×100. Confluent layers of (c) stromal cells and (d) HUVECs. (e) A merged image of (c) and (d). The staggered pillars (white arrows) allow for single layers to be visualized in the combined image. Scale bars = 400 *μ*m. Adapted from Gnecco *et al.*^57^ (f) Cell tracker (red) stained LECs adhering to a lumen with and without the addition of tumor derived fibroblasts (green) at day 0. (g) After 10 days, the lumen reached confluency as shown by F-Actin (red) and nuclear (blue) staining (bottom right). The cells also expressed lymphatic-specific marker Prospero-related homeobox 1 (PROX1; red) and F-Actin (green). Scale bar = 140 *μ*m. Adapted from Lugo-Cintrón *et al.*^64^ (h) A schematic of a microfluidic device for co-culturing blood ECs (red) and LECs (green). (i) Live/dead staining for both blood ECs and LECs. Live cells are stained in green and dead cells are red. (j) All cells were stained with Hoechst (blue), while LECs were stained with lymphatic-specific marker Podoplanin (green) to confirm that LECs were only in the lower channel. Scale bars = 100 *μ*m. Adapted from Sato *et al.*^5^

Co-culture systems can also be used to model *in vivo* systems and diseases. Gnecco *et al.* developed a two layered PDMS microfluidic system that models the endometrium, the inner layer of the uterus [Figs. 4(a)–4(e)].^57^ This system allowed for communication between ECs and stromal cells via paracrine signaling to allow for (1) the stroma cells to differentiate into decidua, (2) cytoskeletal alignment in the ECs due to shear stress, and (3) a sustainable model that lasted up to four weeks, the length of the menstrual cycle. This provides an alternative for animal studies in future trials and is a tool for testing pharmaceuticals. Another system that many investigators try to model is the blood brain barrier (BBB) as it inhibits drug delivery to the central nervous system.^82,83^ Booth and Kim used a microfluidic co-culture model to look at the permeability of various neuroactive drugs, including Gabapentin, Sertraline, and Varenicline.^58^ The model used a PDMS and glass to form two separate channels and a polycarbonate membrane to utilize as a co-culture surface and allow for diffusion. Electrode pads were also embedded on both sides of the membrane to provide trans-endothelial resistance (TEER) measurements. By providing flow, this system allows for measurements while the cells are under shear stress, which can increase TEER.^84^ The co-culture models generally had a higher TEER and lower drug permeability than static models. This study indicates that an *in vitro* BBB model for testing new central nervous system drugs may be feasible.

Motor neuron diseases, like amyotrophic lateral sclerosis (ALS), can be induced by vascular dysfunction. However, they have been difficult to model as animal models are not sufficient. Osaki *et al.* used ECs and motor neuron spheroids to develop a model of EC–neuronal interactions.^59^ A multi-channel chip with motor neuron spheroids and ECs embedded in the collagen gel between channels that provided fresh media. This chip design provided paracrine interactions and perfusion of vessels. Integration of ECs with the neuronal networks caused elongation of the neurites, increased calcium signaling, and upregulated neuronal differentiation. Fluid flow and permeability of the vasculature changed the calcium signaling pattern.

On the lymphatics side, lymphangiogenesis was studied to allow for a better model with interstitial flow included. Kim *et al.* used a fibrin channel with LECs seeded on the outside to interface with the media in the fluid channel and two outside channels containing fibroblasts to provide paracrine factors.^60^ They found that interstitial flow affects the amount, the morphology, and the direction of sprouting lymphatic vasculature. However, it does not play a significant role in the assembly of lymphatic networks. On a cellular level, interstitial flow increases extracellular signal-regulated kinase (ERK) signaling, Prospero-related homeobox-1 (Prox1) expression, and proliferation in LECs.

Since lymphatics are part of the immune system, immune cell–LEC interactions have been studied in microfluidics. Serrano *et al.* designed a chip to replicate the interstitial flow seen in the lymphatic capillaries.^61^ They added peripheral blood mononuclear cells (PBMCs) with and without the addition of the inflammatory cytokine tumor necrosis factor alpha (TNF-α) and found that TNF-α increased PBMC recruitment. In addition, blocking the CXCR4 and CCR7 surface markers, which help attract and traffic immune cells using chemoattractants, significantly decreased PBMC infiltration.

As both blood and lymphatic vasculature play an important role in the transport of nutrients, immune cells, and oxygen, a model for understanding microcirculation is vital. Sato *et al.* used a PET membrane based microfluidic system with both blood ECs and LECs [Figs. 4(h)–4(j)].^5^ The ECs and LECs seeded “back-to-back” on opposite sides of the membrane. The ECs expressed normal markers including VE-Cadherin for both ECs and podoplanin for the LECs. Flow induced junction formation and treatment with histamine increased permeability. This model system could be used to gain a better understanding of the microcirculation system.

Both lymphatic and blood vessels play a key role in cancer progression.^85–87^ Therefore, having microfluidic tumor models with vasculature integrated is vital for cancer research. Buchanan *et al.* grew breast cancer tumor cells and ECs in collagen hydrogels to form a channel based on a method from a previous study.^62,88^ As shear stress increased, the co-culture model down regulated the angiogenic factors produced by the tumor cells. However, the tumor cell monocultures did not see this shift, indicating that the ECs play a major role in this genetic change. Using dextran, the investigators showed that co-culture models appeared to be more permeable compared to monoculture. Kim *et al.* looked at metastatic brain cancer cells while using ECs and astrocytes to model the brain tumor microenvironment.^63^ Cancer cells were suspended in a collagen hydrogel in the center channel of the microfluidic chip and supplemented with media by the channel beside it. Astrocytes in a hydrogel were introduced into a parallel outer channel, and the ECs were grown on the outside of that gel. This design allowed for spatial organization of the various cell types. The use of these cells increased the secretion of inflammatory cytokines serpin E1, interleukin-8 (IL-8), and secreted phosphoprotein 1 (SPP-1). The cancer cells also upregulated the TNF signaling pathway and nuclear factor kappa-light-chain-enhancer of activated B cells (NF-κB). Last, this model was used to test drugs for cancer treatment. They found that the cells were resistant to Palbociclib, which was predicted to be effective based on the sequencing analysis of clinical samples. However, which drugs were effective depended on what type of cancer cells was being used. Models like this system could provide more insight into how drugs react to patient specific cells.

Tumor associated lymphangiogenesis is linked to poor cancer outcomes through invasion into the vasculature and lymph node metastasis.^89^ To gain a better understanding of how lymphatic vessels can promote metastasis, Lugo-Cintrón *et al.* developed a model system that integrated a lymphatic vessel with tumor derived fibroblasts (TDFs) from head and neck cancer patients [Figs. 4(f) and 4(g)].^64^ The system utilized patient derived fibroblasts seeded in a collagen hydrogel surrounding a lumenized lymphatic vessel. The use of a microfluidic chip allowed for perfusion into the gel and lymphatic sprouting. The TDFs caused longer lymphatic sprouts than monoculture or healthy fibroblasts, except in one patient derived sample. The fibroblasts (both healthy and TDFs) saw more sprouting than LECs on their own. The TDFs also caused increased permeability in most of the samples. Among the patient samples, it was also seen that insulin-like growth factor 1 (IGF1) and integrin beta-3 (ITGB3) were upregulated while Serpin E1 and metalloproteinase inhibitor 3 (TIMP3) were downregulated compared to the monoculture. Last, they tested IG-1 as a potential therapeutic. While it did appear to decrease sprout length, number, or permeability for all the patient samples, it was not consistently decreasing all the factors for any patient in the cohort. Gong *et al.* also looked at cancer associated fibroblasts (CAFs) with LECs as well as HUVECs, but from breast cancer instead.^65^ A microfluidic chip was similar in design to the system used by Lugo-Citron *et al.*; however, passive pumping was utilized by making one port larger than the other. The media was perfused approximately two to three times a day, which mimics the flow seen in lymphatic vasculature. They started by showing that the lymphatic vessels had a leakier phenotype than the blood vessels, which is expected based on how much the lymphatic vessels uptake solute. It was found that the CAFs caused an upregulation of tumor and inflammatory factors, including IL-6, IL-8, G-CSF, and HGF. The CAFs also caused decreased barrier function as large amounts of LECs detached from the endothelium. However, cell junction integrity was not noticeably dysregulated. By countering the IL-6 with an antibody, the LECs recovered some of the barrier function. By allowing communication between the LECs and diseased fibroblasts, these papers were able to develop systems that can provide insight into lymphatic dysfunction in cancer and potential systems for testing cancer treatments.

Cho *et al.* looked at the interface between lymphatic vessels, blood vessels, and the tumor environment.^66^ HUVECs and LECs were seeded into channels alongside a collagen hydrogel and allowed to form a monolayer. Then, breast cancer cells were treated with IL-6 to emulate the metastasis process, which stimulated VEGF secretion and lymphangiogenesis. Cancer cells with and without IL-6 were injected into the chip causing the formation of solid tumors on the lymphatic side of the hydrogel. However, invasion behavior was different as the tumors without IL-6 were more rounded, while the ones with IL-6 tended to spread out more. As the tumor colony grew, the HUVECs started to grow toward the lymphatic side specifically targeting the cancer cells.

## ORGAN-ON-A-CHIP

Organ-on-a-chip models are designed to mimic *in vivo* tissue functions to better study disease and physiology compared to animal models and 2D cell culture. Unlike co-culture models, which are just a combination of two or more cell types, organ-on-a-chip models help replicate more complex, tissue or organ level systems and tend to be 3D. Some advancements in these models are included in Table II. With the addition of flow, growth factors, co-culture, and other physiologically relevant features, these models can help test drugs for delivery with patient specific cells, increase the window in which cells are functional, and maintain some of the tissue function that is normally lost *in vitro.*^90^ While the organ-on-a-chip models are more complex, they are not without their limitations. The advantages and disadvantages of this system compared to 2D monoculture and co-culture are included in Table III.

**TABLE III.** Advantages and disadvantages of various culture systems.

Culture type	Advantages	Disadvantages	References
2D Monoculture	Relatively simple Highly studied Relatively inexpensive	Lacks interactions with other cell types Lacks tissue structure Does not reflect *in vivo* morphology Does not provide accurate drug response	91
Co-culture	Shows intercellular interactions Increased complexity to 2D monoculture, but still relatively easy compared to organ-on-a-chip Highly studied Relatively inexpensive	Culture media may be suboptimal for one or more cell lines Difficult to determine where effect comes from (e.g., paracrine signaling vs cell–cell interactions) Can be difficult to distinguish cell lines	92–94
Organ-on-a-chip	Shows intercellular interactions Recapitulates some tissue structure Provides physiological cues Has potential for high throughput drug testing	Less tested than more simple models Difficult to manufacture Takes time to validate Cannot fully recapitulate physiological tissues May not work with standard equipment	90,91,95,96

**FIG. 5.**
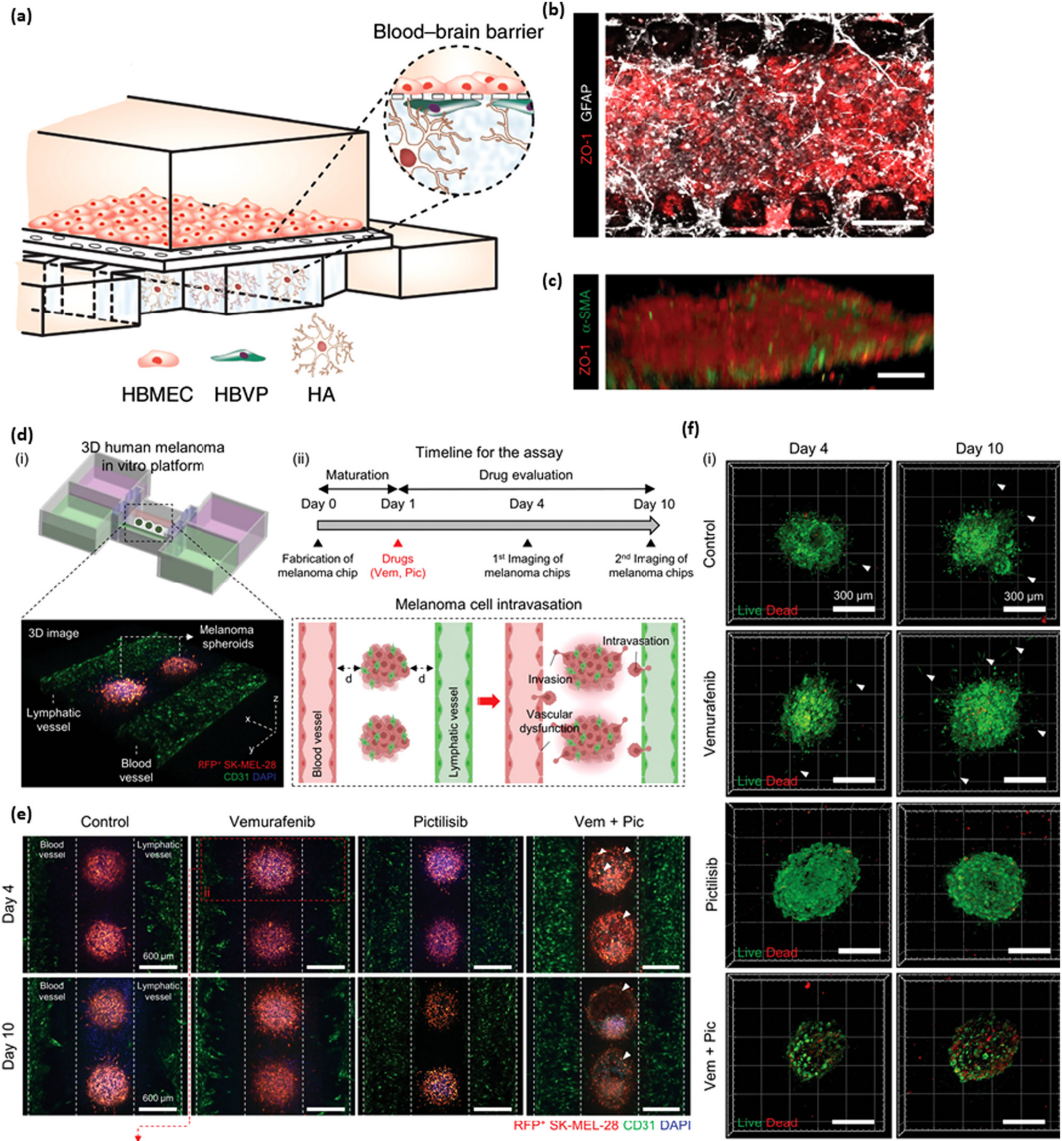
Organ-on-a-chip systems for blood and lymphatic vasculature. (a) A schematic showing the blood brain barrier including ECs, astrocytes, and pericytes. (b) A bottom view of the microfluidic device including an EC monolayer (red) and astrocytes (white). (c) A monolayer of ECs (red) and pericytes (green). Scale bars = 50 *μ*m. Adapted from Ahn *et al.*^62^ [d(i)] A model of an *in vitro* platform with both lymphatic and blood vessels surrounding melanoma spheroids. [d(ii)] The timeline for fabrication, intravasation, treatments, and imaging of the melanoma chips. (e) The spheroids were treated with various BRAF/PI3K inhibitors including 5 *μ*M of vemurafenib, 5 *μ*M pictilisib, and a combination of the two. They were imaged at 4 and 10 days. The spheroids contained RFP positive cells. The vessels were stained with CD31 (green). Scale bar = 600 *μ*m. White arrows indicate where the morphology of the spheroids was dysregulated. (f) Live/dead staining (red and green, respectively) was performed on the spheroids after treatment. Scale bars = 300 *μ*m. Adapted from Cho *et al*.^80^

Organ-on-a-chip models have been used to recapitulate vasculature from many systems including the brain, retina, and gut.^97^ Modeling the BBB in 3D can help to gain a better understanding of how particles move through it and diseases that affect the barrier function. Ahn *et al.* developed a chip with brain microvascular ECs, astrocytes, and pericytes [Figs. 5(a)–5(c)].^67^ This model expressed high amounts of genes associated with BBB specific proteins, helped the astrocytes to maintain their morphology and function, and expressed aquaporin-4, which is vital to regulating homeostasis in the brain. They then used this model to show that the distribution of nanoparticles could be seen at the cellular level and determined that high-density lipoprotein (HDL)-mimetic nanoparticles can be taken up, indicating that they are a potential therapeutic. Organ-on-a-chip models have also been used to model brain diseases, such as Alzheimer's (AD). Shin *et al.* used wild type and familial AD mutation expressing ReNcell, immortalized neural progenitor cells, in a Matrigel system alongside brain ECs to create diseased and healthy models.^68^ The AD model showed an increase in vascular permeability, a decrease in endothelial and tight junction markers, and an increase in reactive oxygen species (ROS), MMP2, and Interferon γ (IFNγ). β-amyloid (Aβ) peptides, which accumulate in AD, were seen to appear on the surface of the brain ECs in the AD model. Treating with an Aβ inhibitor increased the vascular permeability. These models show promise for understanding and treating diseases associated with brain vasculature.

As the outer blood retinal barrier (oBRB) is vital for controlling the movement of solutes and toxins into the eye and contributes to many diseases including age-related macular degeneration and diabetic retinopathy, modeling the oBRB could help gain a better understanding of disease progression.^98^ Chen *et al.* combined HUVECs, fibroblasts, and retinal pigmented epithelial cells (ARPE-19) into microfluidic device integrated with TEER electrodes to mimic the oBRB.^69^ The investigators verified that the ECs were forming vasculature, and that the ARPE-19 cells were placed correctly. While there was variation in the TEER reading, they were able to monitor both cell types together and apart from each other, indicating that after some modification this may be a usable system for monitoring the monolayer. Arik *et al.* also developed an oBRB model but focused more on permeability through Fluorescein angiography and visualization of vasculature.^70^ After treating the vessels with H_2_O_2_ to induce leaking, the vessel permeability increased, but no major damage was done to the ARPE-19. They were able to get optical coherence tomography of the vessels and found it could detect large structural abnormalities as well as microvessels. Both models show promise as oBRB models for detecting permeability and could be developed into disease models.

The gastrointestinal tract vasculature plays a major role in preventing dangerous molecules from damaging other organs.^99^ Dysfunction of this vasculature can lead to disorders such as inflammatory bowel diseases, Celiac disease, and liver disease.^100^ Shin *et al.* designed a human gut model chip to understand how the gut develops.^71^ It was determined that flow through the bottom chamber, under the epithelium, was needed for 3D morphogenesis and stopping the flow decreased villi-like formations. Wnt signaling pathway antagonists were found to decrease in the villi-like structures. The formation of these villi structures is important as they are necessary for a physiologically relevant model. Microfluidics make models like this system possible.

As mentioned in the flow section, lymphangions are the functional units of the lymphatic system. They contain two main components: LECs and lymphatic muscle cells (LMCs). This unit is necessary for fluid drainage. Selahi *et al.* were able to model this system with a lumenized vessel in collagen hydrogels [Figs. 5(d)–5(f)].^72^ After the LMCs were added to the vessel, they migrated toward the LECs to form a gap like what was seen *in vivo*. It was confirmed that the LMCs aligned circumferentially in co-culture. Intermediate shear stress caused poor axial LEC alignment and circumferential LMC alignment. The endothelium was permeability tested and was leakier to small molecules. When treated with TNF-α was added, permeability increased. Overall, this model is tunable, has flow integrated and can be combined with other tools for genetic analysis.

Additionally, there are many lumenized perfusable lymphatic vessel-on-a-chip models. Ilan *et al.* created a 3D lymphatic vessel-on-a-chip to determine how flow and growth factors VEGF-A and VEGF-C affect cellular junctions and lymphatic sprouting.^73^ Regardless of flow type, VEGF-C caused significantly more discontinuities leading to loosened junctions compared to VEGF-A. When all the data points were pooled by flow type without separation based on growth factors, it was found that there was no significant difference between samples treated with no flow, interstitial flow, luminal flow, and both interstitial and luminal flow. When both flow and growth factors were used to separate the data, it was found that treatment with VEGF-C and interstitial flow caused greater discontinuities and button-like junctions. However, this treatment did not induce sprouting. VEGF-A with interstitial flow or interstitial and luminal flow had higher amounts of lymphatic sprouting and zipper-like junctions.

Lymphatic vessels-on-a-chip can also be used to model inflammation and disorders associated with it like lymphedema. Kraus and Lee designed a chip to explore the effects of acute and chronic inflammation on lymphatic vessels.^74^ After using TNF-α to induce inflammation, it was found that acute inflammation led to decreased lymphatic drainage (i.e., uptake of nanospheres) while chronic inflammation had no significant change. This change was found to be associated with changes in fibrillin, an anchoring filament that affects the flap valves along the vessels. When measuring lymphatic permeability using dextran, it was found that chronic inflammation caused a notable increase while acute inflammation did not. This effect is associated with cell–cell junction disruption. Treatment with dexamethasone, an anti-inflammatory, improved the drainage dysfunction caused by acute inflammation but did not significantly help with chronic inflammation. Lee *et al.* utilized a lymphatic vessel-on-a-chip to look at Rho-associated protein kinase (ROCK) in both BECs and LECs affected by inflammation.^75^ ROCK inhibitor was found to improve drainage by loosening junctions between LECs that had been tightened by inflammatory cytokine inflammation. This inflammation and subsequent treatment also caused the opposite effect in blood ECs (i.e., inflammation caused poor barrier function while ROCK inhibition tightened it). By testing ROCK1 and ROCK2, ROCK isoforms, it was found that they are differentially expressed in LECs and blood ECs. ROCK1 inhibition helps restore blood barrier function after cytokine exposure. ROCK2 knockout caused junction loosening in LECs allowing more drainage than those exposed to inflammatory cytokines. Based on these findings, a lymphatic-specific ROCK2 inhibitor was used in an *in vivo* murine lymphedema model. It was found that the ROCK2 inhibitor decreased tail swelling indicating a reversal of the induced lymphedema.

While the lymph nodes (LNs) play a major role in immune cell trafficking, there have not been any lymph node-on-a-chip models that clearly integrate endothelial cells. Most models look at cancer and immune cell integration into the LN.^101–103^ LECs tend to differentially express markers throughout the LN which could make modeling the endothelium a challenge.^104^ For example, caveolin 1 appears in ceiling LECs in the subcapsular sinus, but tumor necrosis factor receptor superfamily member 9 (TNFRSF9) was seen in the floor LECs. In the future, the LN should be modeled to gain a better understanding of the lymphatic system.

Organ-on-a-chip models can also be used to model tumors. Nguyen *et al.* utilized it to model pancreatic ductal adenocarcinoma vascularization and found that these tumors can remove the endothelium to create tumor luminal structures.^76^ The proposed mechanism for this ablation is activin-ALK7 signaling. Spheroids are a common mechanism for modeling tumors as they provide a heterogeneous environment. Wan *et al.* created vascularized tumor spheroids with multiple methods.^77^ They focused on three main methods: tumor cells alone, tumor cells and fibroblasts mixed, and tumor cells followed by fibroblasts. The final method, sequential, appeared to be the most effective, which was supported by an improvement in perfusion. Sequential tumor spheroids also caused higher amounts of T cell recruitment when subjected to continuous flow for four days. Lymphatic vasculature has also been used in conjunction with tumor spheroids. Frenkel *et al.* saw lymphatic vasculature sprouting increased in both area and length in response to the colon cancer spheroids.^78^ The LECs maintained their EC and lymphatic markers despite their close interactions with the cancer spheroids. Cho *et al.* saw an increase in CCL21 in the lymphatic sprouts when using breast cancer spheroids,^79^ indicating that CCL21-CCR7 signaling is implicated in tumor metastasis. Overall, these models allow for modeling cancer metastasis via blood and lymphatic vessels.

As both blood and lymphatic vasculature are implicated in cancer metastasis, models have been developed with both types of ECs. Cao *et al.* used bioprinted blood and lymphatic vessels with tumor cells seeded between them for both perfusion and drainage.^80^ They started by verifying that permeability values were like native vessels. Then they looked at the transport of drug molecules. The addition of draining lymphatic vasculature increased diffusion and increased viability in cancer cells when Doxorubicin, an anti-cancer drug, was added. This may be due to the removal of the drug through lymphatic drainage. Cho *et al.* also used bioprinted vessels but looked at melanoma instead of breast cancer.^81^ The spheroids were placed between the vessels. BRAF/PI3K inhibitors, which had been shown to decrease fibroblast-assisted invasion and growth, were administered through the blood vessels. The spheroids were destroyed by day 10 and both vessels experienced less invasion. However, the vessels were dysregulated, and the ECs were detached. These studies allow for the integration of both perfusion and drainage to make a more physiologically relevant model for testing cancer drugs.

## CONCLUSIONS

In this article, systems with increasing complexity for modeling lymphatic and blood vasculature have been highlighted. Microfluidic systems allow for the growth of small vessels with the integration of other factors including flow, ion concentration, and other cell types. They offer powerful new tools to test new drugs and gain a better understanding of vessel development. While microfluidics platforms provide more physiologically relevant models compared to monoculture, there are still many aspects that need to be explored including the integration of multiple organ systems together to look at how organs affect each other and the use of diseased endothelial cells to look at more vascular disorders. In addition, the integration of different types of vessels (e.g., arterioles or collecting vessels) with the capillaries within these microfluidic chips could provide more insight into how the vasculature functions in various states. As stated previously, the ECs within the LNs need to be modeled further as it would be beneficial for drug discovery, understanding immune cell trafficking, and cancer progression. However, there are challenges. Variability in cell marker expression in different vasculature can make it difficult to model.

In comparison to *in vivo* models, microfluidic chips are limited to the chemical cues provided by the selected cell lines seeded on the chip and can be missing essential crosstalk from peripheral tissues. However, recent vascular-related studies have demonstrated that certain cell types in microfluidic devices can display *in vivo* properties through supplemental addition of factors.^71^ Furthermore, there are limitations in the physical properties (i.e., material composition, potential fabrication artifacts, etc.) of microfluidic devices that impact their stability in long-term experiments.^5^ Another potential complication for the widespread adoption of microfluidics is that many of these systems are not standardized making it costly in both time and labor. Further technological advancements would be necessary to improve the usage time and manufacturing of microfluidic chips. While *in vivo* models offer great features relating to environmental signaling and aging, microfluidic systems provide insight into fluidic flow that greatly impacts cellular response and adaptivity. Overall, microfluidics is taking promising steps toward more complicated, controllable models for both blood and lymphatic vasculature.

## Data Availability

Data sharing is not applicable to this article as no new data were created or analyzed in this study.
